# Crier versus Hope and Currie

**Published:** 1906-03-17

**Authors:** 


### CRIER VERSUS HOPE AND CURRIE.

The case of Crier and wife versus Hope and Currie, which occupied Mr. Justice A. T. Lawrence and a special jury during four days of last week, is one of such signal interest and importance to the medical profession as to call for more than a bare report of the proceedings and of the result. Drs. Currie and Hope are medical practitioners in partnership at Hanwell and West Ealing; and the plaintiffs, who were comparatively new residents in the district, sued them for alleged negligence on the part of Dr. Currie, whereby he was said to have communicated the infection of scarlet fever to the female plaintiff. The case was curiously complicated by the circumstance that Drs. Hope and Currie, although partners, were not on speaking terms, and that some litigation was pending between them in respect of their partnership relations. Dr. Hope, moreover, was parish doctor and medical officer of health for the district; and, in the latter capacity, came into official relations with the patients of Dr. Currie, when that gentleman had notified that they were the subjects of contagious disease. Such was the state of things when Dr. Currie was requested to attend Mrs. Crier, the plaintiff, in her approaching confinement. On November 14, 1904, Dr. Currie was asked to see the child of a Mrs. Miller, apparently a person in humble circumstances. He pronounced it to be suffering from scarlet fever, notified the case, and had no more to do with it, but he observed that a second child in the same house was ailing. On the 15th he was asked to see the second child also, and notified it as another case of scarlet fever. He walked home, and on arrival found a telegram calling him to Mrs. Crier. According to his own evidence, he went direct to his bathroom, disinfected his hands and arms with lysol, changed his coat, and put on another reserved for obstetrical cases. He then visited other patients, again returned home, walked half a mile to a cab rank, and drove to Mr. Crier's house. After speaking to the patient, and apparently shaking hands with her, he went to another room, took off his coat, scrubbed his arms and hands with soap and water with a special obstetrical nail-brush, then used corrosive sublimate, then lysol again, then put on india-rubber sleeves, previously disinfected, made an examination, and went downstairs to await the progress of events. When recalled to the patient he went through the same disinfecting process as before, and remained with her until her delivery. All went well until the 18th, when the lady's temperature rose to 105°, and on the following day both she and her nurse suspected that she had scarlet fever, a belief which Dr. Currie confirmed on the 20th. Scarlet fever was present in the neighbourhood at the time; but Dr. Currie told Mr. Crier that he had not "attended" any cases; apparently attaching no importance to the two single visits to the two Miller children. Unfortunately, however, Dr. Hope, who called at Mr. Crier's house in his official capacity as medical officer of health, told Mr. Crier that he was Dr. Currie's partner, and that Dr. Currie had attended cases of the fever. On this, Dr. Currie was dismissed from farther attendance upon Mrs. Crier, and other gentlemen were called in. She had a lingering illness, complicated with white leg and a pelvic abscess, and was described at the trial by the plaintiff, her husband, as a "wreck." The action was brought to recover damages generally, for negligence, and a sum of £260 was claimed for special damage in the way of expenses out of pocket incurred. Dr. Hope was made a defendant by reason of his legal liability for the acts of his partner; and the action was defended by the Medical Protection Society.

No one who reads the whole of this most regrettable story can fail to perceive that the plaintiffs had sustained a great misfortune, or can wonder at their endeavour to obtain redress, an endeavour clearly stimulated by Dr. Currie's original denial of his attendance upon scarlet fever, a denial which was afterwards found to be inconsistent with the facts. But the plaintiff's chance of obtaining a verdict was practically extinguished by the first words of the learned judge, who told the jury that it rested with the plaintiffs to make out their case, or, in other words, to show that the scarlet fever had been communicated to the plaintiff by the defendant. Of course there was not, and from the nature of the case there could not be, sufficient evidence to prove that the scarlet fever was communicated to the plaintiff by Dr. Currie; and the probability would be that it reached her through some other and possibly quite unsuspected channel. The disease was prevalent at Ealing, and the possible channels of transmission would in such circumstances be countless.

The case establishes that a medical practitioner, who takes reasonable and proper precautions to avoid the carrying or dissemination of infection, will be exempt from responsibility for accidents which he could neither foresee nor prevent.

